# 1418. The Effect of Dry Hydrogen Peroxide on Environmental Contamination with *Candida auris* in an Adult Burn Unit

**DOI:** 10.1093/ofid/ofad500.1255

**Published:** 2023-11-27

**Authors:** Jared Alan Sutton, Nychie Dotson, Jaquelyn Nagy, Julia Moody, Kenneth E Sands

**Affiliations:** HCA Florida Blake Hospital, Bradenton, Florida; HCA Healthcare West Florida Division, Tampa, Florida; HCA Florida Blake Hospital, Bradenton, Florida; HCA Healthcare, Nashville, Tennessee; HCA Healthcare, Nashville, Tennessee

## Abstract

**Background:**

*Candida auris* (*C. auris*) is an emerging multi-drug resistant pathogen that is a health threat for hospitalized patients. *C. auris* may survive on surfaces where standard disinfectants may not achieve adequate disinfection. Continuous dry hydrogen peroxide (DHP) reduces microbial bioburden on surfaces and may reduce the risk of C. *auris* transmission in the clinical care setting. This study reviews the efficacy of DHP exposure, coupled with routine environmental cleaning, on reducing microbial burden of *C. auris* on surfaces.

**Methods:**

DHP-emitting systems were installed on burn intensive care and burn stepdown units. After install, for each patient newly identified with *C. auris*, baseline surface sampling of the patient room and other unit areas was performed beginning on the first day of positive culture, defined as Day 0. The DHP systems were activated and surface sampling collected to include days 1, 7, and 14. Select surfaces were sampled using polyurethane foam neutralizing buffer swabs. Individual swabs were grouped into three composites: Inner Patient Halo, Outer Patient Halo, and Mobile Shared Equipment (Table 1). Bioburden was assessed through cultures of the swabs and the identification and enumeration of resulting colony forming units (CFU). A multivariate regression model was used to evaluate the potential relationship between duration of DHP exposure and the logarithmically transformed counts of *C. auris* yielded by the samples.

**Table 1:**
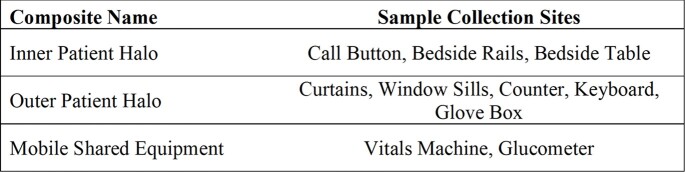
Sampling site locations included in each composite category

**Results:**

Three patients were identified, resulting in collection of 36 composite swabs. *C. auris* CFU were obtained from all composite swabs, with observed decrease in count over time (Figure 1). In a regression model including included covariates for time, room and patient proximity, there was a statistically significant relationship between *C. auris* bioburden and exposure to DHP (Table 2, F(5,30)=6.999, p< 0.001, adjusted R^2^=0.461), with duration of DHP exposure adding significantly to the relationship, p=0.

**Figure 1:**
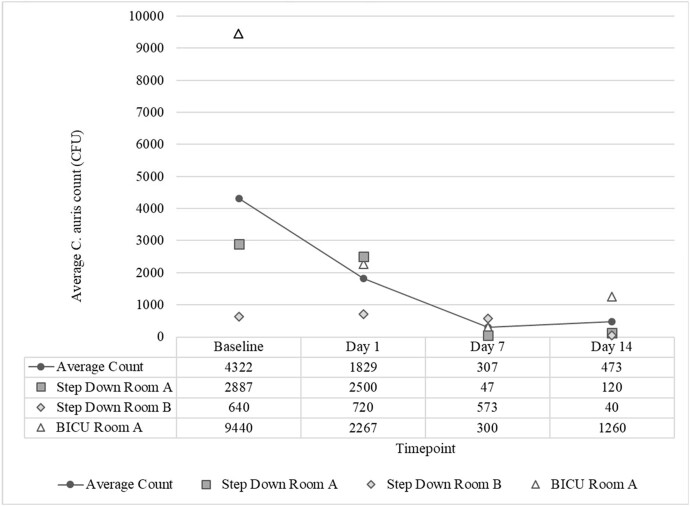
Candida auris colony forming units enumerated from environmental surfaces.

**Table 2:**
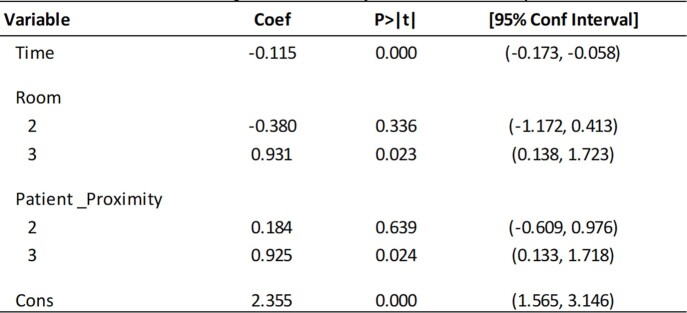
Multivariate Regression Analysis of DHP’s Impact on Candida auris Bioburden

**Conclusion:**

Continuous DHP, coupled with routine environmental cleaning, augments disinfection and is effective in reducing surface microbial bioburden in rooms of patients with *C. auris* colonization/infection. To fully understand the synergism between routine environmental cleaning and DHP, further research is needed.

**Disclosures:**

**All Authors**: No reported disclosures

